# Implementation of SAMPL guidelines: Recommendations for improving statistical reporting in biomedical journals

**DOI:** 10.1016/j.clinme.2025.100304

**Published:** 2025-03-27

**Authors:** Michal Ordak

**Affiliations:** Department of Pharmacotherapy and Pharmaceutical Care, Faculty of Pharmacy, Medical University of Warsaw, Banacha 1 Str., Warsaw 02-097, Poland

**Keywords:** Clinical medicine, SAMPL guidelines, Biostatistics, Statistical reviews, Statistical analysis

## Abstract

The quality of statistical reporting in biomedical journals remains insufficient despite the introduction of SAMPL guidelines in 2015. These guidelines aim to improve clarity and accuracy but are underutilised by authors and editorial boards. Common deficiencies include unclear descriptions of statistical test purposes, inadequate reporting of effect sizes, poor analysis of assumptions, and limited consideration of outliers. Addressing these challenges requires broader adoption of SAMPL recommendations, improved statistical literacy among researchers and editors, and stronger editorial oversight. To enhance transparency and reliability in biomedical research, the SAMPL guidelines should become standard practice, supported by targeted training and clear guidance for authors.

## Introduction

In 2015, the SAMPL (Statistical Analyses and Methods in the Published Literature) guidelines were introduced to improve the clarity and accuracy of statistical reporting in biomedical research. These guidelines provide detailed recommendations on how to report basic statistical methods, including the description of statistical tests, the interpretation of results, and the reporting of effect sizes and assumptions. Their primary goal is to reduce common errors in statistical reporting that can undermine the reliability and reproducibility of research findings.^1^ This article reflects on common deficiencies in statistical reporting and how the SAMPL guidelines can address these challenges. Despite their potential, SAMPL guidelines remain underutilised, with many journals failing to integrate them into workflows.^2^ For instance, only 5% of the 141 members of the World Association of Medical Editors (WAME) actively reference these guidelines in their practices.^3^ The lack of implementation of these guidelines leads to persistent issues in statistical reporting, including, for example, unclear justification of statistical tests, omission of practical significance measures, and failure to address assumptions or outliers.^4^ The review process, based on SAMPL guidelines, highlighted recurring issues such as insufficient descriptions of statistical methods, inadequate attention to assumptions, and failure to report effect sizes. In my experience as a statistical reviewer and editor, addressing these gaps not only improved the quality of individual manuscripts but also underscored a deeper challenge – the lack of familiarity with basic statistical principles among many authors. Authors frequently overlook fundamental concepts outlined in SAMPL, which often results in revisions that must address basic statistical shortcomings. This points to a broader need for education and proactive engagement with SAMPL guidelines, which, if implemented widely, could elevate the overall standard of statistical reporting in biomedical research. Despite some improvements, SAMPL guidelines have not been universally adopted, reflecting a broader challenge in biomedical publishing: the gap between awareness and action. While editors and authors may acknowledge the value of these guidelines, their practical application often falls short due to a lack of systematic implementation and accountability. Placing these guidelines on the WAME website is a step in the right direction,^3^ but it is insufficient to ensure their proper implementation. Improving statistical reporting requires more than acknowledging SAMPL guidelines – it demands their active integration into journal workflows, supported by clearer instructions for authors and targeted training for editors and reviewers. Addressing these challenges through practical editorial strategies and broader educational efforts could significantly elevate the quality and transparency of statistical reporting in biomedical research.

## Statistical reporting and the role of SAMPL guidelines

In my experience as a statistical reviewer and editor, I have observed that many biomedical manuscripts suffer from recurring issues in statistical reporting. These include vague descriptions of statistical methods, insufficient assessment of test assumptions, lack of clarity in reporting software and effect sizes, and a general underappreciation of outliers or missing data. Authors also frequently misinterpret results or use inappropriate post-hoc tests, which compromises the validity of their findings. Applying the SAMPL guidelines in statistical review has proven to be an effective tool in addressing these shortcomings. When authors are provided with clear, structured feedback based on these guidelines, the quality of reporting tends to improve significantly. The iterative nature of the review process allows for refining both the clarity and accuracy of statistical presentation. Incorporating SAMPL recommendations not only strengthens individual manuscripts but also contributes to greater transparency, reproducibility, and trust in biomedical research overall.

## Recommendations

### Include SAMPL guidelines in instructions for authors

Making SAMPL guidelines accessible to authors is a key step in improving statistical reporting. Encouraging WAME members to include SAMPL guidelines in instructions aligns with raising editorial standards.^5^ By integrating these guidelines, journals can set clear expectations for authors, encouraging them to follow best practices in statistical reporting. Furthermore, editorial boards should familiarise themselves with these guidelines to ensure consistent enforcement during the review process.

### Require author declarations in cover letters

Ensuring that authors actively engage with SAMPL guidelines can prevent common errors from the outset. Authors should be required to declare in their cover letters that they have familiarised themselves with SAMPL guidelines and applied them during their statistical analyses. This additional step ensures that authors acknowledge the importance of adhering to basic statistical principles. Journals could implement policies where manuscripts without such a declaration do not proceed to the peer-review stage. This simple yet effective approach would emphasise the importance of statistical rigor from the outset. Nicholas, in his 2019 published article, pointed out that the cover letter should contain key information related to submitting the manuscript to a specific journal.^6^

### Organise online training for editorial boards

Editors play a critical role in ensuring the quality of statistical reporting. Regular training sessions for editorial board members could significantly improve the enforcement of SAMPL guidelines. During these sessions, common statistical errors made by authors could be discussed, along with practical ways to address them. Examples from journals like *JAMA* and the *World Journal of Diabetes* illustrate the effectiveness of such meetings, where editors focus on the correctness of statistical analyses as a key criterion for manuscript evaluation.^7^^,^^8^ Online sessions could also foster discussions about challenges in implementing SAMPL guidelines, helping editors build consensus on best practices.

### Advocate for SAMPL guidelines at international events

Scientific conferences provide an excellent platform to promote the adoption of SAMPL guidelines. Presenting the rationale for implementing SAMPL guidelines at prominent events, such as the World Conference on Research Integrity (WCRI) and the International Congress on Peer Review and Scientific Publication, would further promote their adoption. These events provide platforms for discussing practical recommendations to improve statistical reporting and foster collaborations among editors, reviewers and researchers.^9^^,^^10^ Highlighting the impact of SAMPL guidelines during such conferences could encourage more journals to integrate them into their workflows.

### Promote active engagement with SAMPL guidelines

For SAMPL guidelines to be effective, journals must go beyond passive acknowledgment. A passive approach to implementing SAMPL guidelines, such as merely referencing them on journal websites, is unlikely to yield significant results. Instead, journals must actively engage with these guidelines by:

• requiring compliance with SAMPL in all submitted manuscripts
• providing clear examples in the instructions for authors to illustrate common statistical errors and their corrections
• encouraging reviewers to explicitly reference SAMPL guidelines in their feedback to authors.

## Conclusion

The SAMPL guidelines offer a practical and urgently needed solution to longstanding issues in statistical reporting. Yet, their limited adoption underscores a pressing challenge: the gap between acknowledging the importance of statistical rigor and embedding it into daily editorial practices. This article highlights how applying SAMPL guidelines during statistical reviews can lead to significant improvements, not only in individual manuscripts but in the overall integrity of biomedical research. To achieve this, a collective, committed effort is essential. Journals must actively integrate SAMPL guidelines into their workflows to make them a standard practice. Embedding them into instructions for authors, requiring author declarations, organising training for editorial boards, and promoting their importance at international conferences are all necessary steps. By taking these actions, journals can set a new standard for statistical reporting, one that prioritises integrity, reproducibility, and the credibility of published research. Implementing SAMPL guidelines is a shared responsibility. Editors, reviewers, and organisations like WAME must lead by embedding these principles into workflows. Without this, the credibility and reproducibility of biomedical research will remain at risk.

## Funding

This research did not receive any specific grant from funding agencies in the public, commercial, or not-for-profit sectors.

## CRediT authorship contribution statement

**Michal Ordak:** Writing – review & editing, Writing – original draft, Methodology, Formal analysis, Data curation, Conceptualization.

## Declaration of competing interest

The author declares that there are no conflicts of interest.
